# Chancery Lunatics

**Published:** 1853-07-01

**Authors:** 


### CHANCERY LUNATICS.

*Return to an Order of the Honourable the House of Commons, dated 28 February,* 1853 *;—for,*

A Return, "showing the number of Lunatics against whom Commissions of Lunacy are now in force ; total amount of incomes ; sums allowed for maintenance ; amount of income ; allowance for maintenance according to scales of income : also, amount of per-centage, 1839 to 1852, on Lunatics' incomes received under the Act 3 & 4 Will. IV., c. 36, and payments made thereout (in continuation of Parliamentary paper, No. 378, of Session 1839)."

---

A Return, showing the number of Lunatics against whom Commissions of Lunacy are now in force; total amount of incomes; sums allowed for maintenance; amount of income; and allowance for maintenance according to scales of income.

| | | Income. £ s. d. | Maintenance. £ s. d. |
|---|---|---|---|
| There are 514 | persons against whom Commissions of Lunacy are now in force. | | |
| | The total of whose annual incomes amounts to . | 281,907 13 6 | |
| | And the total of the sums allowed for their maintenance, to . . . . . . . | | 177,825 8 5 |
| Of the above 514 | persons, there are— | | |
| 99 | who individually have less than 100*l.* per annum, and whose incomes together amount to . . . | 5,846 14 7 | |
| | And the total of the sums allowed for their maintenance to . . . . . . . | | 5,679 6 8 |
| 117 | who individually have more than 100*l.*, and less than 200*l.* per annum, and whose incomes together amount to . . . | 17,441 10 11 | |
| | And the total of the sums allowed for their maintenance to . . . . . . . | | 15,238 19 11 |
| 94 | who individually have more than 200*l.*, and less than 400*l.* per annum, and whose income together amount to . . . | 26,536 8 0 | |
| | And the total of the sums allowed for their maintenance to . . . . . . . | | 21,748 2 4 |
| 56 | who individually have more than 400*l.*, and less than | | |
| | Carried forward . . | £49,824 13 6 | £42,666 8 11 |

| | | Income. | Maintenance. |
|---|---|---|---|
| | Brought forward . | £49,824 13 6 | £42,666 8 11 |
| | 600*l.* per annum, and whose incomes together amount to . . . | 27,348 0 0 | |
| | And the total of the sums allowed for their maintenance to . . . . . . | | 21,870 11 6 |
| 47 | who individually have more than 600*l.*, and less than 1000*l.* per annum, and whose incomes together amount to . . . | 35,695 0 0 | |
| | And the total of the sums allowed for their maintenance to . . . . . . | | 23,480 16 0 |
| 65 | who individually have more than 1000*l.*, per annum, and whose incomes together amount to . . | 169,040 0 0 | |
| | And the total of the sums allowed for their maintenance to . . . . . . | | 89,807 12 0 |
| 36. | In 36 of the above-mentioned 514 cases, the incomes have not yet been ascertained, nor the allowances for maintenance fixed, chiefly on account of the recent date of the Commissions in such cases. | | |
| 514 | | £281,907 13 6 | 177,825 8 5 |

(Signed) CHARLES N. WILDE,

Registrar in Lunacy.

11 April, 1853.

---

Amount of per centage, 1839 to 1853, on Lunatics' Incomes, received under the Act 3 & 4 Will. IV., c. 36, and payments made thereout (in continuation of Parliamentary paper, No. 378, of Session 1839).

---

Amount of per centage of one per cent. on the Incomes of Lunatics, received by the Bank of England, with the privity of the Accountant-General of the Court of Chancery, to "The Account of the Board of Visitors for the better care and treatment of Lunatics;" under the provisions of the said Act, viz.:

| | £ | s. | d. |
|---|---|---|---|
| From 8 January 1839 to 8 January 1840 . . | 3,288 | 8 | 8 |
| " . . 1840 . " . 1841 . . | 2,676 | 9 | 0 |
| " . . 1841 . " . 1842 . . | 2,316 | 15 | 11 |
| " . . 1842 . " . 1843 . . | 2,519 | 16 | 0 |
| " . . 1843 . " . 1844 . . | 2,196 | 14 | 8 |
| " . . 1844 . " . 1845 . . | 2,654 | 6 | 5 |

| | | | | £ | s. | d. |
|---|---|---|---|---|---|---|
| From 8 January | 1845 | to 8 January | 1846 . . | 3,193 | 4 | 0 |
| „ . . | 1846 . | „ . | 1847 . . | 3,741 | 19 | 7 |
| „ . . | 1847 . | „ . | 1848 . . | 3,722 | 19 | 2 |
| „ . . | 1848 . | „ . | 1849 . . | 3,143 | 6 | 0 |

*Note.*—In addition to the per-centage on the incomes of Lunatics, paid pursuant to the said Act, there have been paid into the Bank of England, in the name of the said Accountant-General, to the credit of the account above mentioned (pursuant to several orders of the Lord Chancellor), the following sums, being the amounts of surplus fees taken in the office of the Secretary of Lunatics, beyond the sums required for the salaries and expenses of such office, namely:

| | | £ | s. | d. |
|---|---|---|---|---|
| 1839 . . | 29 April . . | 2,262 | 0 | 8 |
| 1841 . . | 1 April . . | 1,205 | 14 | 3 |
| 1841 . . | 27 August . . | 404 | 18 | 7 |
| | Total . . | £3,872 | 13 | 6 |

No payment has been made into the above-mentioned account, in respect of the fees taken in the office of the Secretary of Lunatics, since the payment on the 27th August, 1841. The fees taken in the office of the Secretary of Lunatics have, since then, been paid into the Suitors' Fee Fund Account, pursuant to the Act 6 Vict. c. 84.

---

Amount of the payments made by the Bank of England, with the privity of the said Accountant-General, out of the said account for the salaries and expenses under the said Act, viz.:

| | | | | £ | s. | d. |
|---|---|---|---|---|---|---|
| From 8 January | 1839 | to 8 January | 1840 . . | 5,931 | 10 | 0 |
| „ . . | 1840 . | „ . | 1841 . . | 2,401 | 18 | 0 |
| „ . . | 1841 . | „ . | 1842 . . | 2,672 | 10 | 0 |
| „ . . | 1842 . | „ . | 1843 . . | 2,718 | 10 | 0 |
| „ . . | 1843 . | „ . | 1844 . . | 2,702 | 16 | 0 |
| „ . . | 1844 . | „ . | 1845 . . | 3,268 | 14 | 0 |
| „ . . | 1845 . | „ . | 1846 . . | 3,043 | 8 | 0 |
| „ . . | 1846 . | „ . | 1847 . . | 3,009 | 16 | 0 |
| „ . . | 1847 . | „ . | 1848 . . | 2,839 | 10 | 0 |
| „ . . | 1848 . | „ . | 1849 . . | 2,756 | 0 | 0 |

*Note.*—The payments made as above-mentioned, between the 8th day of January, 1839, and the 8th day of January, 1840, amounting together to 5,931*l.* 10*s.*, included the salaries and expenses of nine quarters; such salaries and expenses having, prior to the 8th day of January, 1839, been in arrear, owing to the insufficiency of the fund to discharge the payments becoming due under the Act.

The accounts of the above receipts and payments have been annually taken and allowed by the Master in Chancery pursuant to the provisions of the said Act.

The foregoing particulars were included in a return, dated the 25th June, 1851, made pursuant to an order of the House, dated the 7th August, 1850.

Account of Receipts and Payments, continued for the subsequent period.

*Receipts.*

| | | | | | £ | s. | d. |
|---|---|---|---|---|---|---|---|
| From 8 January | 1849 | to 8 January | 1850 | . . | 3,640 | 16 | 7 |
| ,, . . | 1850 . | ,, . | 1851 | . . | 4,477 | 5 | 6 |
| ,, . . | 1851 . | ,, . | 1852 | . . | 3,097 | 19 | 3 |
| ,, . . | 1852 . | ,, . | 1853 | . . | 3,828 | 8 | 2 |

*Payments.*

| | | | | | £ | s. | d. |
|---|---|---|---|---|---|---|---|
| From 8 January | 1849 | to 8 January | 1850 | . . | 2,776 | 4 | 0 |
| ,, . . | 1850 . | ,, . | 1851 | . . | 2,786 | 4 | 0 |
| ,, . . | 1851 . | ,, . | 1852 | . . | 3,359 | 0 | 0 |
| ,, . . | 1852 . | ,, . | 1853 | . . | 2,734 | 12 | 0 |

*Note.*—In addition to the above payments, the above-mentioned sum of 3,872*l.* 13*s.* 6*d.*, was on the 28th June, 1852, pursuant to an order of the Lord Chancellor, transferred from the said per-centage account to the Suitors' Fee Fund Account of the Court of Chancery.

(Signed) H. Enfield,
8 April, 1853. Secretary to the Visitors of Lunatics.

